# Deep Learning-Based Detection, Classification, and Segmentation of Cerebral Microbleeds

**DOI:** 10.5152/eurasianjmed.2026.261414

**Published:** 2026-07-16

**Authors:** Habip Eser Akkaya, Önder Polat, Hasan Çolakoğlu, Dorukan İnanç Akpınar, Erhan Kaya

**Affiliations:** 1 Department of Radiology, Ankara Training and Research Hospital, Ankara, Türkiye; 2 Department of Electrical and Electronics Engineering, Gaziantep Islam, Science and Technology University, Gaziantep, Türkiye; 3 Department of Radiology, Ümraniye Training and Research Hospital, İstanbul, Türkiye; 4 Department of Public Health, Kahramanmaraş Sütçü İmam University, Kahramanmaraş, Türkiye

**Keywords:** Artificial intelligence, brain, cerebral microbleeds, deep learning, microhemorrhages

## Abstract

**Background:**

Artificial intelligence (AI) has increasingly been integrated into various fields of medicine, necessitating rigorous oversight by scientists to ensure its appropriate application. This study presents an analysis of microhemorrhages, which are closely associated with significant cerebrovascular events. Researchers have designed a study to investigate the feasibility of utilizing AI in the detection and assessment of microhemorrhages.

**Methods:**

The study retrospectively analyzed 108 patients with cerebral microhemorrhagic foci and 108 controls using cranial magnetic resonance imaging (MRI) and susceptibility-weighted sequences, following ethical approval. Deep learning models, including a customized DenseNet for classification and nnU-Net for segmentation, were trained on preprocessed MRI data to detect and localize microhemorrhages efficiently.

**Results:**

The study involved a total of 216 patients with and without cerebral microbleeds. There were a total of 146 foci in the 108 patient group. The classification model demonstrated an area under the curve of 80% ± 2.7% and an accuracy of 75% ± 1.7%, though specificity was more limited at 68% ± 4.3%. The segmentation model identified microhemorrhagic regions with a sensitivity of 89.4% ± 1.4% and achieved a Dice similarity coefficient of 62.05% ± 2.3%.

**Conclusion:**

Overall, both models demonstrated the potential for detecting and localizing microhemorrhages in 3D MRI data, though the classification model showed moderate specificity. The findings suggest that AI-based approaches may eventually serve as a supportive tool for microhemorrhage detection, provided further refinements are made to enhance detection accuracy and clinical applicability.

**Main Points**

- Deep learning models demonstrate promising performance in the detection and localization of cerebral microbleeds on magnetic resonance imaging.
- While the classification and segmentation models offer robust discriminative capacity and high sensitivity, there remains potential for improvement in terms of specificity and overall performance.
- Artificial intelligence–based approaches demonstrate potential as supportive tools in the detection of microbleeds.

## Introduction

Small hemorrhagic lesions, with a maximum size of 10 mm, detected using susceptibility-weighted (SWI) magnetic resonance (MRI) imaging are referred to as cerebral microhemorrhages or, occasionally, cerebral microbleeds (CMBs).^1^

Histopathologically, CMBs are associated with hemosiderin deposition resulting from the breakdown of blood products leaking from small vessels. They typically appear as small, round, homogeneous, hypointense foci on T2-weighted gradient-recalled echo sequences.^2^^,^^3^Cerebral microbleeds are minute chronic brain hemorrhages that are common in numerous conditions such as stroke, neurodegenerative disorders, traumatic brain injury, as well as following radiation therapy for brain and head and neck malignancies.^4^

Cerebral microbleeds are involved in pathological processes affecting the brain’s small vessels, such as hypertensive vasculopathy and cerebral amyloid angiopathy.^2^ Their detection and interpretation have gained increasing significance due to the aging population.^3^ They are not easily detected using conventional computed tomography or MRI scans, highlighting the necessity for standardized diagnostic methods to enhance their identification.^5^

Artificial intelligence (AI) comprises computer software that employs complex mathematical algorithms to process input data and generate predefined outputs, aiming to simulate human intelligence. The adoption of AI has expanded significantly in recent years, particularly with the emergence of deep learning, which has facilitated the development of AI models with significantly more layers than were previously feasible.^6^^,^^7^

The field of radiology has played a pioneering role in integrating AI into medical practice, thereby advancing medicine into the digital age. While billions of digitally archived radiological images provide a robust foundation for AI research, the full potential of AI in radiology remains an area of active investigation.^7^

This study aimed to utilize machine learning techniques to analyze CMBs in cranial SWI sequences. This research has the potential to contribute to patient care and scientific advancements by enabling the rapid and objective detection of CMBs, ultimately aiding in diagnosis, treatment, prognosis, and the prevention of complications in the aging population amid the rapid progression of technological advancements.

## Material and Methods

**Figure 1.**
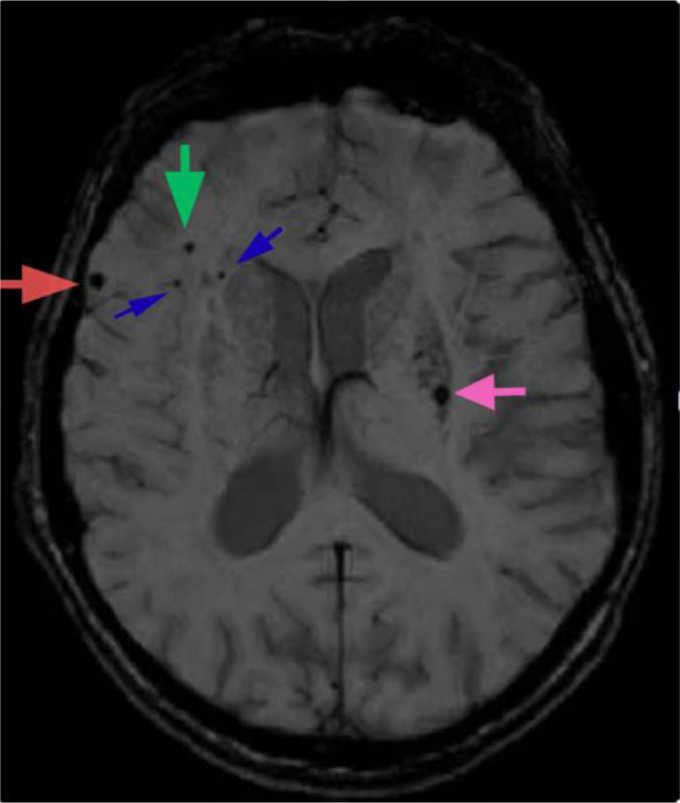
Susceptibility-related microhemorrhagic foci are seen in the cortical, subcortical, and deep white matter of the right frontal lobe and in the left basal ganglia.

After obtaining approval from the ethics committee of Ankara Training and Research Hospital hospital (E-93471371-514.99-223933196, 07.09.2023), patients with cranial MRI and SWI sequences between January 2021 and May 2023 were reviewed retrospectively from the hospital system. A signed consent form was obtained from the individuals stating that they agreed to participate in the study. There were 127 patients aged 0-99 years with microhemorrhagic foci. Patients with a history of previous cranial surgery (5 patients) and trauma-related hemorrhage (14 patients) were excluded from the study. Cerebral microbleeds were included in 108 patients. As a control group, 108 individuals aged 0-99 years with no previous history of cranial surgery were included in the study (Figure 1). For classification, each MRI was labeled as patient or not. For segmentation, an expert neuroradiologist marked the parts with CMBs on the MRI and mask images were obtained to be used in the deep learning model. For both tasks, the models were trained using a laptop with Nvidia RTX3060 GPU with 6 GB VRAM.

### Magnetic Resonance Imaging Acquisition Technique

For all patients, MRI scans were performed using a 1.5 Tesla (Siemens Aera, Erlangen, Germany) device available in the department. The routine sequences applied for brain MRIs included sagittal T1-weighted, transverse T1-weighted, transverse T2-weighted, transverse fluid-attenuated inversion recovery, and diffusion-weighted images. In cases where pathology was suspected during routine imaging, additional post-contrast transverse and coronal T1 sequences were acquired following the administration of a contrast agent (intravenous gadolinium at a dose of 0.1 mmol/kg). Table 1 presents the parameters used for SWI in this study. The evaluations were conducted retrospectively by reviewing the cases on the picture archiving and communication system monitors and recording the findings.

### Statistical Analysis

Statistical analyses were performed using IBM SPSS Statistics for Windows, version 22.0 (IBM Corp.; Armonk, NY, USA). Mean and SDs were calculated for the descriptive analysis, and categorical variables are represented as numbers and percentages.

### Data Preparation and Preprocessing

To ensure consistent input for the deep learning models, several preprocessing steps were applied to the brain MRI scans. For both the classification and segmentation tasks, the intensity of all pixel values was scaled to the range (0, 1). Additionally, foreground regions were cropped to remove black background areas, focusing the model’s attention on the regions of interest.

For the classification task, each MRI slice was resized to a fixed dimension of 128×128 to create uniform input sizes for the deep learning model. This dimension was selected to standardize input for the DenseNet model while maintaining a balance between computational load and the retention of diagnostic features. Unlike patch-based methods, which focus on isolated high-resolution regions, this whole-slice approach allows the model to process the entire anatomical context. This global view is crucial for the classification task, as it enables the network to differentiate actual microbleeds from similar-looking vascular structures, calcifications, or imaging artifacts by analyzing their relationship to surrounding tissues, a distinction that is often lost when looking at small, isolated patches.

For the segmentation task, 3D patches of size 64 × 64 × 16 were cropped from the MRI volumes. To maximize training efficiency, 8 patches were extracted from each MRI volume. Positive patches, which contained microhemorrhages, were cropped with the lesion positioned at the center of the patch. This ensured that the model focused on learning relevant features in the context of the lesion. Negative patches, which did not contain microhemorrhages, were also included to balance the dataset and improve the model’s robustness.

### Deep Learning Models

This study utilized convolutional neural network (CNN) architectures for both classification and segmentation tasks, with a specific focus on optimizing performance for the challenging problem of detecting microhemorrhages. The classification task aimed to identify whether a given MRI scan contained microhemorrhages, while the segmentation task focused on localizing them within the scans.

For classification, a custom-designed DenseNet^8^ architecture was implemented, which builds on the principles of densely connected convolutional networks but was tailored to the specific characteristics of the dataset and problem at hand. For segmentation, the nnU-Net model was utilized. An overview of the DenseNet architecture and a description of the modifications made to suit the requirements of this study are provided below.

### DenseNet Architecture for Classification

To classify whether an MRI contains microhemorrhages, a customized 3D DenseNet model was employed. DenseNet, short for Densely Connected Convolutional Network, introduces direct connections between all layers within each dense block. This structure improves information flow and gradient propagation during training, enabling the network to achieve better performance with fewer parameters compared to traditional architectures.

### Customized DenseNet Model

**Figure 2.**
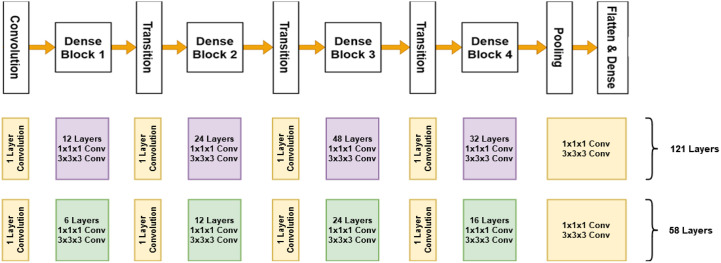
DenseNet 121 layers and 58 layers architecture.

The modified DenseNet architecture diverges from the standard DenseNet-121 in several ways, particularly in the growth rate and the number of layers in each dense block. The details of the architecture are described below:

1. Dense Block Configuration
  In the standard DenseNet-121, the dense block configuration is (6, 12, 24, 16), meaning the first dense block has 6 layers, the second has 12 layers, and so on. In contrast, the customized model uses a smaller configuration of (3, 6, 12, 8). This reduces the number of layers in the first and final dense blocks, which effectively reduces the overall model depth and computational cost (Figure 2).
2. Growth Rate
  The growth rate, which defines the number of feature maps added by each layer within a dense block, is set to 16 in the model compared to 32 in DenseNet-121. A smaller growth rate results in fewer feature maps, further reducing the number of parameters while still preserving the dense connectivity that facilitates feature reuse.
3. Total Number of Parameters
  Due to the smaller block configuration and reduced growth rate, the customized DenseNet architecture has significantly fewer parameters than DenseNet-121, which has approximately 7 million parameters. The reduced complexity makes the model more suitable for training on medical imaging datasets similar to ours, which often have limited samples.
4. Bottleneck Layers
  Bottleneck layers, which are standard in DenseNet architectures, compress the feature maps before they are passed to the dense layers. The bottleneck size in the model is consistent with DenseNet-121, ensuring computational efficiency without sacrificing performance.

By reducing the block configuration and growth rate, the customized DenseNet model achieves a good balance between computational efficiency and representational power. The architecture leverages the dense connectivity of DenseNet to extract meaningful features across the spatial dimensions of the MRIs, which is crucial for the classification of subtle abnormalities such as microhemorrhages. This modification to the DenseNet architecture was implemented to ensure the model could be trained within the hardware constraints of a 6 GB VRAM GPU. Furthermore, by reducing the depth and width of the network, the total number of trainable parameters was significantly lowered. This simplified architecture acts as a form of regularization, helping to prevent overfitting, a common challenge when training deep learning models on smaller medical datasets where the sample size is limited.

### Segmentation Task and nnU-Net Architecture

In medical imaging, segmentation tasks involve delineating specific regions of interest within an image, such as pathological areas, organs, or tissues. For this study, segmentation was used to localize microhemorrhages in 3D brain MRI scans. Segmentation provides pixel-level annotations, making it a critical step for understanding the spatial distribution of microhemorrhages and assisting in clinical decision-making.

To perform this task, the nnU-Net architecture was utilized,^9^ a self-configuring deep learning framework specifically designed for medical image segmentation. nnU-Net builds upon the widely recognized U-Net architecture, a fully convolutional network known for its symmetric encoder-decoder structure with skip connections. nnU-Net extends the U-Net design by dynamically adapting its hyperparameters, preprocessing steps, and network configurations to the specific dataset being used. This adaptability eliminates the need for extensive manual tuning, making nnU-Net a robust choice for diverse medical imaging tasks.

**Figure 3.**
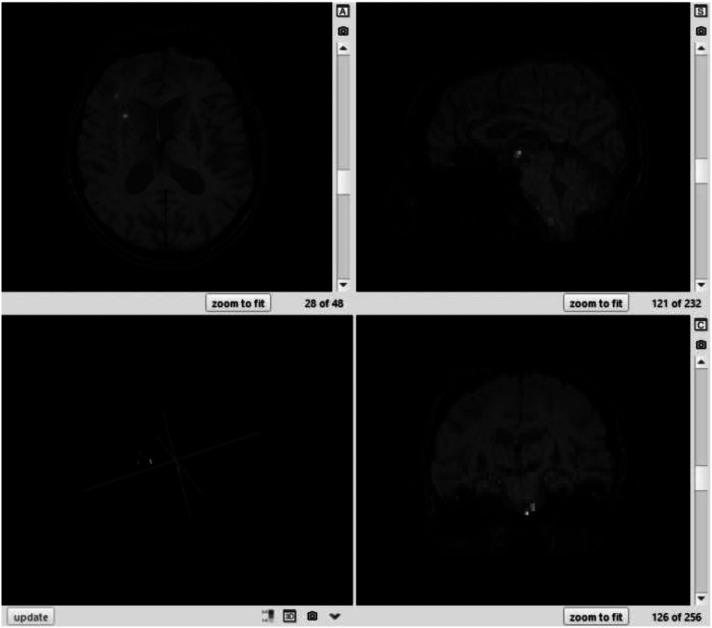
Representative multiplanar ITK-SNAP view demonstrating manual placement of color-coded anatomical landmarks on axial magnetic resonance imaging with corresponding sagittal, coronal, and 3D spatial coordinate visualization.

In this study, nnU-Net was trained using ground truth masks generated by an expert neuroradiologist, who carefully labeled the microhemorrhages in the MRI volumes using the ITK-SNAP software.^10^(Figure 3). These masks served as the target output for the segmentation model, enabling it to learn to accurately localize microhemorrhages within the 3D MRI data. By combining nnU-Net’s advanced features with expert annotations, the segmentation model achieved precise and reliable performance in detecting microhemorrhages.

### Model Training and Hyperparameters

To ensure the reproducibility of the findings, the specific training configurations for both deep learning architectures are detailed in Table 2. The classification model was developed using the DenseNet architecture, optimized with the Adam algorithm to minimize binary cross-entropy loss. For the segmentation task, the nnU-Net framework was employed, utilizing a compound loss function combining Dice loss and cross-entropy to effectively handle the class imbalance typical in microbleed segmentation.

## Results

Of the 108 patients with CMBs, 55 were female (50.9%) and 53 were male (49.1%). The mean age of the individuals was 59.88 ± 18.91 years. Of the 108 patients without CMBs, 53 were female (49.1%) and 55 were male (50.9%). The mean age of the individuals was 57.75 ± 15.73 years.

The total number of microhemorrhagic foci in patients with CMBs was 146. For classification, the model was aimed to recognize whether a patient in a given MRI image had CMBs, whereas for segmentation, the model was aimed to identify whether there were microhemorrhagic foci in 3D patches taken from a given MRI image. The performance of the models was evaluated using a repeated random subsampling validation strategy over 5 independent iterations. This approach differs from conventional k-fold cross-validation; instead of dividing the data into fixed groups, the entire dataset was randomly reshuffled and split for every iteration. This approach was chosen over conventional k-fold cross-validation to enhance methodological transparency and statistical reliability. While k-fold cross-validation partitions data into fixed, non-overlapping folds, repeated random subsampling allows for the dataset to be reshuffled and split anew for every iteration, ensuring that each training and testing cycle is entirely independent. For each iteration, the data were distributed into 3 subsets: training set (60%) used to develop the model’s primary detection capabilities, validation set (15%) used for hyperparameter tuning and early stopping to prevent overfitting, and held-out test set (25%) used to calculate final performance metrics. Following each iteration, the model was reset and retrained from scratch on a new random distribution of data. All performance metrics reported in the following sections represent the mean and SD derived from the 5 independent test sets. This rigorous approach ensures that the reported effectiveness of the AI is not biased by any single specific data split.

### Classification Results

**Figure 4.**
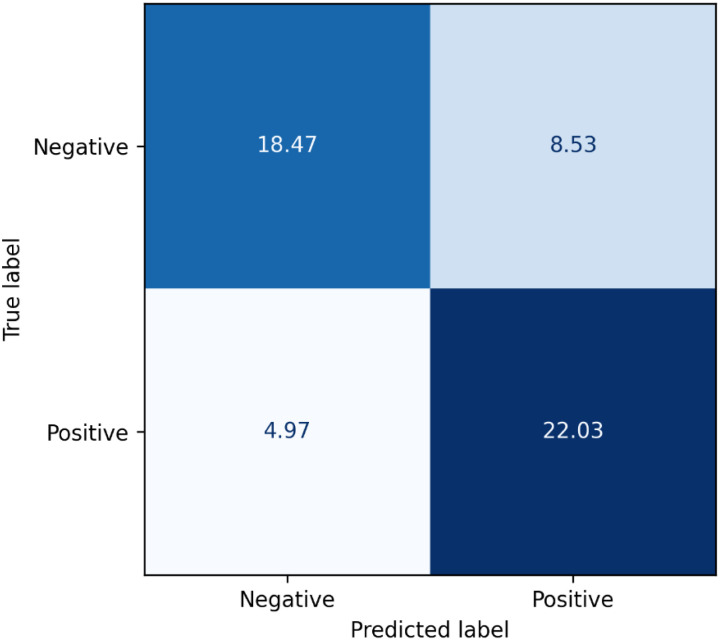
Confusion matrix for classification. Values represent the average number of patients obtained from 5 random iterations.

The classification model achieved an area under the curve (AUC) of 80% ± 2.7%, indicating an ability to distinguish between MRIs with and without microhemorrhages. The accuracy (ACC) of the model was 75% ± 1.7%, demonstrating its overall effectiveness in correctly classifying MRIs. The model also exhibited a sensitivity of 81.6%, meaning it correctly identified microhemorrhages in approximately 81.6% ± 1.8% of the instances where they were present. However, the specificity of the classification model was 68% ± 4.3%, indicating that the model’s ability to correctly identify MRIs without microhemorrhages was somewhat lower. The performance results and the corresponding confusion matrix are shown in Table 3, and Figure 4, respectively. All metrics were reported as the average of the 5 iterations.

### Segmentation Results

**Figure 5.**
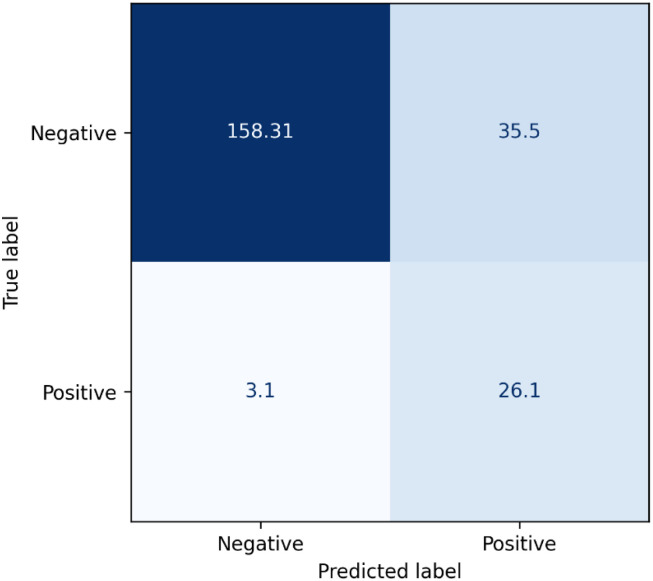
Confusion matrix for segmentation. Values represent the average number of patients obtained from 5 random iterations.

For the segmentation task, the sensitivity score was computed based on the model’s ability to correctly identify patches containing microhemorrhages on the test data. The segmentation model achieved a promising sensitivity of 89.4% ± 1.4% and a dice similarity coefficient (DSC) of 62.05%, successfully identifying and localizing microhemorrhagic regions within MRI patches. Other metrics are presented in Table 4 and the confusion matrix for the segmentation model is given in Figure 5. Note that 8 patches were selected for each MRI volume. In the selection process, all microhemorrhages were included and the rest of them were chosen from the volumes without any microhemorrhages (negative patches). All metrics were reported as the average of the 5 iterations. For the segmentation task, the nnU-Net architecture effectively localized microhemorrhages. The model demonstrated a sensitivity of 89.4% ± 1.4% for patch-level detection and a DSC of 62.05% for pixel-level spatial overlap. This performance is consistent with the inherent challenges of segmenting extremely small, sparse lesions like cerebral microbleeds, where minor boundary variations significantly influence overlap metrics

In summary, both classification and segmentation models performed well in detecting microhemorrhages, with the classification model achieving a good AUC and sensitivity, and the segmentation model showing promising sensitivity in identifying microhemorrhages within patches.

## Discussion

Our study group consisted of 108 patients with a mean age of 57.7 years, with a nearly equal gender distribution. A total of 146 microhemorrhagic foci were detected. Overall, while the classification model demonstrated promising AUC and sensitivity, the segmentation model accurately identified microhemorrhages with good precision. The classification model applied to categorize microhemorrhages achieved an AUC of 80% ± 2.7% and an accuracy of 75% ± 1.7%. Sensitivity was measured at 81.6%, while specificity was 68% ± 4.3%. In the segmentation analysis, where 8 patches were selected per MRI volume, the model demonstrated a sensitivity of 89.4% ± 1.4% in detecting microhemorrhagic patches. Accuracy was recorded at 82.7%, specificity at 81.7%, and AUC at 83.8%. The good accuracy rates found in both models highlight an important step toward expanding the use of AI in radiology. The discrepancy between high patch-level sensitivity (89.4%) and the pixel-level Dice score (0.6205) highlights the difference between lesion detection and precise boundary delineation. For targets as small as cerebral microbleeds, the DSC is highly sensitive to minimal spatial shifts. A DSC of 0.6205 indicates that while the model is highly reliable at locating the foci, the exact morphological segmentation remains a challenge, a finding that aligns with current state-of-the-art results in small-vessel disease imaging.

A limitation of the current classification approach is the resizing of MRI slices to 128×128. Since cerebral microbleeds (CMBs) are typically smaller than 10 mm, aggressive downsampling risks the loss of fine-grained morphological details or the blurring of very small foci. While the model achieved a good AUC score, future iterations could utilize high-resolution architectures or hybrid padding techniques to ensure that the smallest lesions are preserved without loss of pixel information.

CMBs are predominantly observed in individuals with cerebrovascular disease and dementia; however, they are increasingly detected in neurological imaging even during normal aging. In recent years, advancements in MRI techniques and their application in elderly populations have led to an increase in CMB detection.^3^ However, studies have shown variations in the number of detected microbleeds, with different prevalence rates observed across similar study groups. Epidemiological studies report a prevalence of CMBs ranging from 5% to 23%. Even when study populations are isolated, such discrepancies have been attributed to differences in MRI protocols. Various technical factors, including scanning methodology, repetition time (TR), echo time (TE), flip angle, bandwidth, pulse-sequence parameters, slice thickness, spatial resolution, and magnetic field strength, play a crucial role in these variations.^1^^,^^11^^-^^13^ These findings underscore the need for a standardized and highly accurate diagnostic method for CMB detection. While the study demonstrated AI’s effectiveness in identifying the presence and location of microhemorrhages, uncertainties remain regarding the extent and manner in which clinicians should incorporate AI-driven findings into their evaluations.

In the field of neurology, AI research has primarily focused on brain tumors and stroke cases. Sensitivity and specificity rates for large vessel occlusion detection have been reported to range from 76% to 94%. For example, in a multi-center study utilizing deep learning for large vessel occlusion detection, AI demonstrated a sensitivity of 85.8% and a specificity of 96.9%. For isolated M2 segment occlusion of the middle cerebral artery, AI software achieved 69% sensitivity and 96% specificity.^14^^,^^15^

Various studies have previously explored microbleed detection using different AI methodologies. A two-stage study analyzed 1843 CMBs in 393 patients using quantitative susceptibility mapping (QSM) and deep learning. In Stage 1, the 2.5D fast radial symmetry transform algorithm was combined with a 1-layer convolutional network to detect potential CMB locations in QSM images. In Stage 2, the V-Net model was used to eliminate false positives. This study demonstrated a high sensitivity of up to 94.9% in Stage 1 and 93.5% in Stage 2, with an overall sensitivity of 88.9%.^16^ Another study implemented a 3D deep residual network for brain microbleed detection, training, and testing on 7T SWI data from 73 individuals with radiation-induced microbleeds. This model achieved a detection sensitivity of 71.9% in 12 test patients (4). A comprehensive study analyzing 320 MRI scans with a 3D fully convolutional network (3D FCN) strategy reported a high sensitivity of 93.16%, with an average of 2.74 false positives per subject.^17^

Duan et al examined the reliability and accuracy of disease area segmentation using a deep learning system (DLS) with anonymized data from 1500 patients across 12 hospitals. The study used the Dice score to evaluate segmentation performance, comparing AI predictions with those of 6 experienced clinicians. The results demonstrated that a properly trained DLS achieved a Dice accuracy of 0.598 during the training phase for all 4 classes in 30 patients, compared to 0.576 for physicians.^18^ In Liu et al’s study, a deep learning model trained with 154 datasets and tested with 41 cases using 3D SWI and high-pass filtered phase images demonstrated 95.8% sensitivity, 70.9% precision, and 1.6 false positives per case.^19^ The study results align with previous research in the field of microhemorrhage detection. The reported DSC of 62.05% is highly competitive when compared to established benchmarks in the field. For instance, Duan et al reported a DSC of 0.598,^18^ and recent work by Fan et al on CMB segmentation reported a DSC of 0.72 for their 3D-CNN model.^20^ In this context, a DSC exceeding 0.60 indicates that the model is not only reliable at locating the foci but also provides a level of segmentation accuracy that is comparable to the current state-of-the-art results for small-vessel disease imaging. However, the specificity achieved by the classification model was relatively low. Lower specificity may lead to some images without microhemorrhages being incorrectly classified as positive. In clinical practice, such false-positive findings may require more detailed evaluation of otherwise normal images and may lead to additional image review by radiologists. This situation could potentially increase reporting time and contribute to a higher clinical workload. In some cases, false-positive detections may also prompt closer scrutiny of specific anatomical regions or additional follow-up evaluations to exclude true pathology. Therefore, the outputs of the model should be interpreted cautiously within the clinical context and should not be considered as a stand-alone diagnostic tool. Instead, the model is more appropriately used as a supportive decision-assistance system that may help draw attention to suspicious regions while leaving the final interpretation to the radiologist. Although relatively lower selectivity in the classification model was observed, the good sensitivity and accuracy rates suggest that AI-based approaches could contribute valuable insights to scientific literature, researchers, and clinicians. It is believed that each study employing different methodologies in a given field is instrumental in refining and identifying more accurate and applicable techniques. Variations in results can be influenced by factors such as study population, imaging techniques, AI model selection, and numerous other variables.

The proposed machine learning model is intended to function as a supportive decision-assistance tool in clinical practice rather than a stand-alone diagnostic method. In routine radiology, the detection of CMBs is often a non-trivial task that is time-consuming and highly susceptible to human oversight, particularly when lesions are sparse or very small. By integrating this model into the clinical workflow, several realistic scenarios emerge where AI can provide significant utility. In high-volume imaging centers, the model can act as a triage tool by automatically pre-scanning MRI volumes and flagging suspicious regions for the radiologist, thereby directing attention to potential pathologies and mitigating the risk of oversight caused by diagnostic fatigue. Furthermore, due to its promising sensitivity, the model may contribute to the early detection of potential lesions in aging populations, supporting more proactive clinical decision-making. Most importantly, the model offers a systematic framework for longitudinal patient follow-up or comparative analysis of images acquired at different time points. Comparing microhemorrhage counts across multi-year intervals is notoriously difficult for human readers; this model enables a more objective and reliable assessment of lesion progression or the development of new microhemorrhages over time. However, given the moderate specificity observed, the model is most appropriately used as a supportive system that draws attention to suspicious regions while leaving the final pathological verification and interpretation to the radiologist.

### Limitations

Several limitations regarding the dataset must be emphasized. First, the study population was limited to a single center, and the deep learning models were trained on a relatively small dataset. The limited number of MRI scans potentially restricts the model’s ability to generalize to unseen cases, particularly those involving variations in image quality or acquisition parameters. Secondly, this study lacks external validation using an independent dataset from a separate institution. The absence of multi-center data means that the generalizability of the models to different MRI scanners and imaging protocols remains unestablished. Because the models were developed using a standardized imaging protocol from a single institution, they may be highly susceptible to domain shift. In such scenarios, model performance often degrades significantly when encountering MRI scans from different manufacturers or centers with varying signal-to-noise ratios and contrast characteristics. While the use of data augmentation and internal validation strategies helped mitigate overfitting, these results should be viewed as a preliminary proof-of-concept. Future research utilizing larger, multi-ethnic, and multi-center datasets is strictly required to ensure that these models maintain their accuracy and clinical utility across diverse healthcare environments. Third, the training process was constrained by the computational power of the utilized hardware. The limited GPU resources restricted the complexity of the models, the batch size, and the extent of hyperparameter tuning, potentially affecting performance. Longer training times also hindered the ability to explore more advanced architectures or extensive data augmentation techniques. Utilizing more powerful hardware could enable the development of more sophisticated models with improved accuracy and generalizability.

A limitation of the current classification approach is the resizing of MRI slices to 128 × 128. Since CMBs are typically smaller than 10 mm, aggressive downsampling risks the loss of fine-grained morphological details or the blurring of very small foci. This global resizing strategy was chosen over a patch-based approach for classification specifically to preserve the global spatial context necessary for artifact rejection. However, it is recognized that this necessitates a trade-off in resolution. Future iterations could utilize high-resolution architectures, such as vision transformers or hybrid padding techniques, to ensure that the smallest lesions are preserved with their full pixel information while still maintaining the broad field of view required for accurate clinical interpretation.

## Conclusion

The significance of AI in clinical practice extends beyond diagnosis, encompassing the entire disease management process. Artificial intelligence can be utilized not only for predicting and managing complications but also for analyzing data in areas clinicians may not focus on, thereby detecting early signs and issuing alerts.^15^ The study demonstrated that AI-based models can identify and localize microhemorrhages with promising sensitivity, though current specificity and accuracy levels indicate they are best suited as supportive decision-assistance systems rather than stand-alone diagnostic tools. As AI capabilities continue to advance, further research with larger datasets is necessary to improve performance and address the ethical considerations associated with its integration into clinical practice.

## Figures and Tables

**Table 1.** Technical Parameters of Susceptibility-Weighted Imaging

Parameter	Value
Echo time	40 ms
Repetition time	29 ms
Slice thickness	0.7 mm
Flip angle	25
Field of view	185 × 200 mm
Intersection gap	0 mm
Matrix size	320 × 250

**Table 2.** Technical Hyperparameters and Training Configurations

Hyperparameter	Classification (DenseNet)	Segmentation (nnU-Net)
Optimization algorithm	Adam	Adam
Initial learning rate	1 × 10^−4^	1 × 10^−3^
Batch size	4	8 (patch based)
Loss function	Binary cross-entropy	Dice loss + cross entropy
Epochs (Max)	150	150
Input dimension	128 × 128 × N (Slices)	64 × 64 × 16

BCE, binary cross-entropy.

**Table 3.** Performance Metrics for the Trained Classification Model on the Test Set (Mean ± SD) Across 5 Random Test-Set Iterations)

Accuracy (%)	Sensitivity (%)	Specifity (%)	AUC (%)
75.0 ± 1.7	81.6 ± 1.8	68.0 ± 4.3	80.0 ± 2.7

AUC, area under the curve.

**Table 4.** Performance Metrics for the Trained Segmentation Model on the Test Set (Mean ± SD Across 5 Random Test-Set Iterations)

Accuracy (%)	Sensitivity (%)	Specifity (%)	AUC (%)	DSC (%)
82.7 ± 1.5	89.4 ± 1.4	81.7 ± 1.6	83.8 ± 3.4	62.05 ± 2.3

AUC, area under the curve; DSC, dice similarity coefficient.
